# Equal Representation Does Not Mean Equal Opportunity: Women Academics Perceive a Thicker Glass Ceiling in Social and Behavioral Fields Than in the Natural Sciences and Economics

**DOI:** 10.3389/fpsyg.2022.790211

**Published:** 2022-03-16

**Authors:** Ruth van Veelen, Belle Derks

**Affiliations:** Department of Social, Health and Organizational Psychology, Faculty of Social and Behavioural Sciences, Utrecht University, Utrecht, Netherlands

**Keywords:** women in academia, perceived glass ceiling, gender inequality, social identity, career advancement

## Abstract

In the study of women in academia, the focus is often particularly on women’s stark underrepresentation in the math-intensive fields of natural sciences, technology, and economics (NTE). In the non-math-intensive of fields life, social and behavioral (LSB) sciences, gender issues are seemingly less at stake because, on average, women are well-represented. However, in the current study, we demonstrate that equal gender representation in LSB disciplines does not guarantee women’s equal opportunity to advance to full professorship—to the contrary. With a cross-sectional survey among *N* = 2,109 academics at mid-level careers (i.e., assistant and associate professors) in the Netherlands, we test the hypothesis that in LSB (more than NTE), female academics perceive to hit a “thicker” glass ceiling—that is, they see a sharper contrast between the high representation of women at the lower compared to the top levels. We test whether this predicts female academics’ lower estimated chances to reach full professorship relative to men in LSB (but not NTE). We introduce a novel perceived glass ceiling index (GCI), calculated based on academics’ perceptions of the share of women and men in their direct work environment minus their perceptions of gender ratio among full professors in their field. Results confirm that the perceived glass ceiling is thicker in the non-math-intensive LSB compared to math-intensive NTE fields. Furthermore, only in LSB (but not NTE), women perceived a thicker glass ceiling than men. Moreover, only among female academics, the thicker the perceived glass ceiling, the lower their estimated chances to become full professor 1 day. Combined, a moderated mediation showed that for women only, a thicker perceived glass ceiling in LSB compared to NTE disciplines predicted their lower estimated chances to advance to full professor level. No such mediation occurred for men. We conclude that women’s higher numerical representation in LSB disciplines does not negate a male-dominant normative standard about academic leadership and success. Paradoxically, the perceived odds for female academics to reach the top of their field are lower in fields where they are relatively highly represented, and this may pose unique barriers to women’s perceived opportunities for career success.

## Introduction

### Problem Definition

While women are obtaining academic degrees at greater proportions than ever before (54% of BSc/MSc students and 48% of PhD’s in the EU are women), they remain vastly underrepresented in math-intensive fields of Natural Science, Technology^1^ and Economics (NTE; European Commission, 2019; Catalyst, 2020). Without question, this is problematic for gender parity and diversity in these fields. A large body of work has thus already examined the causes and consequences of women’s minority position in NTE fields, such as economics, engineering, and computer science (e.g., Cheryan et al., 2009; Cech, 2015; Hall et al., 2015; Fouad et al., 2016). By contrast, women make up a large and growing proportion of the non-math-intensive fields of Life, Social and Behavioral sciences (LSB). As such, gender issues are seemingly less at stake—and therefore less studied—in LSB disciplines because *on average* gender parity is achieved. Importantly however, following Ceci et al.’s (2014) seminal article on women’s representation across the academic sciences, a complex picture emerges when breaking down the representation of women in math-intensive NTE versus non-math-intensive LSB fields at different career stages. In math-intensive NTE fields, we see a vast underrepresentation of women already at the undergraduate level (≈30% bachelor level) which remains relatively constant further up the ranks in the academic hierarchy (≈25% assistant professors). Yet we see quite a different picture for the LSB fields, such as psychology, where women are heavily overrepresented at an undergraduate level (>70%) and then are less well-represented with every step up in academic rank (≈50% assistant professor level), ultimately ending up a minority at the leadership level (<30% full professor level; Ceci et al., 2014).

The phenomenon whereby women’s odds to advance to higher positions in the organizational hierarchy are lower than men’s is called the *glass ceiling effect* (e.g., Cotter et al., 2001; Elacqua et al., 2009; Kulik and Rae, 2019). The metaphor of a glass ceiling stands for a barrier that is difficult to detect but that nevertheless limits opportunities to climb the organizational ladder. In the current research, we investigate academics’ *perceptions* of a glass ceiling in NTE versus LSB disciplines with the aim to deepen our understanding of how women (compared to men) academics at mid-level careers perceive the social hierarchy in LSB (compared to NTE) fields. We expect women in LSB fields to see a starker contrast in women’s overrepresentation at the lower, yet underrepresentation at the top levels in the hierarchy compared to in NTE fields. We expect the perception of a “thicker” glass ceiling in LSB to lower women’s (but not men’s) perceived opportunities to attain leadership themselves some day. That is, we test the premise that in LSB fields where—on average—gender parity is achieved, a thicker perceived glass ceiling poses a unique barrier for women’s upward career mobility toward academic leadership that, paradoxically, we may not observe in the male-dominated NTE fields.

### Research Goals

Prior US studies show that while only few women opt for a math-intensive NTE education, once they are “in” the glass ceiling they face in advancing their academic careers is relatively thin, at least until the assistant professor level (Ceci et al., 2014; Miller and Wai, 2015). Since the 1990s the odds for women in the US in math-intensive studies to advance from bachelor to PhD level are similar to men’s (Miller and Wai, 2015). Since 2007, the odds for women in math-intensive NTE fields to advance from PhD to assistant professor level are also similar to men’s (Ceci et al., 2014). Yet to the contrary, in non-math-intensive LSB fields the odds for women to proceed from PhD to assistant professorship are significantly lower (22 percent points) compared to men’s (Ceci et al., 2014). This supports the idea that while well-represented at the undergraduate and early career level, women in LSB fields are likely to face a thick glass ceiling in advancing their academic careers toward leadership.

Expanding from the US studies described above, the current study focuses on glass ceiling effects in academia in the Netherlands. Our target population is further up the career ladder, namely, mid-level career academics (i.e., assistant and associate professors) and their perceptions about career advancement to leadership (i.e., full professorship). Following Ceci et al. (2014), we focus on three academic fields that can be categorized as math-intensive (Natural Sciences, Technology and Economics; NTE) and three fields that can be characterized as less math-intensive (Life, Social and Behavioral Sciences; LSB). Mid-level career academics are sampled from all 14 universities in the Netherlands (N ≈ 2000). The Netherlands ranks relatively low on the representation of women in academic leadership compared to other European countries (European Commission, 2019). In 2017 (the year of data collection in this study) a mere 21% of full professors in the Netherlands were women (LNVH, 2018). Moreover, investigations from over 10 years ago show that, on average, women in Dutch academia had lower promotion probabilities than men, particularly at the highest academic ranks (e.g., Groeneveld et al., 2012). Yet in the Dutch context, research offering a disaggregated view on gender differences in promotion probabilities across academic fields is, to our knowledge, largely absent.

**Figure 1:**
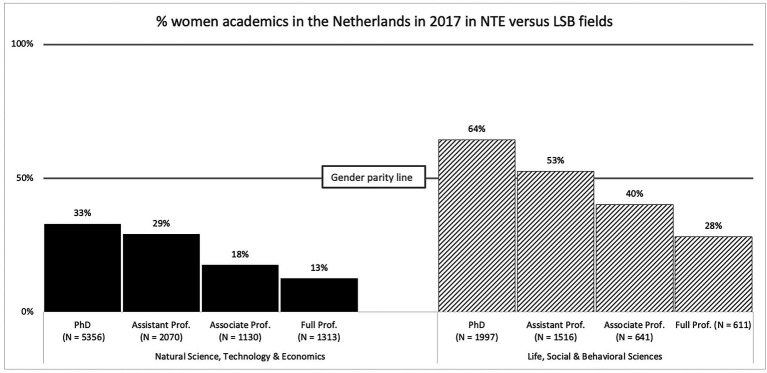
Percentage (%) women academics in the Netherlands in 2017, per scientific discipline [total numbers per Rank × Field displayed below the bars (*N*)]. Note: Based on VSNU/WOPI data 2017.

In Figure 1, a graph is displayed with the representation of women (in %) per NTE and LSB field and per academic rank in the Netherlands in 2017. With each step higher in academic rank, the representation of female academics is lower, with female full professors ultimately being a minority in all fields. Although the percentage of female full professors is higher in the LSB compared to the NTE fields, and relatively, women’s drop in representation with every step up in rank is comparable, the psychological meaning of this drop may be different in the LSB compared to the NTE disciplines. To illustrate, in NTE fields women are underrepresented at all levels, making up about 1/3rd of academics at PhD (33%) and assistant professor (29%) level and 18% (associate professor) and 13% (full professor) further up the career ladder. By contrast, women in LSB disciplines are still (slightly) overrepresented at PhD (64%) and assistant professor level (53%) yet drop vastly under the gender parity line at associate (40%) and full professor (28% level).

Moving beyond attempts to locate or identify an “actual” glass ceiling (Cotter et al., 2001), this research examines the glass ceiling as a social construct, described by the term *perceived glass ceiling* (e.g., Foley et al., 2002). Female academics in NTE fields are likely to perceive women to be a minority overall, irrespective of their status position in the academic hierarchy. Female academics in LSB fields, however, are likely to see that women are overall well-represented, yet *not* in leadership positions, thus seeing a gender inequality in positions of high status. We take a socio-psychological approach and rely on social role and social identity theory (Tajfel and Turner, 1979; Eagly, 1987) as a novel theoretical approach to understand these glass ceiling perceptions. Specifically, we argue that when women see that their very group membership as a woman puts them at risk for facing barriers in upward mobility, this has negative consequences for their perceived future career prospects. To this end, investigating *perceptions* of a glass ceiling is important because when women perceive that a glass ceiling exists (i.e., perceive that men have more access to higher status positions than women) they may also be less likely to pursue career promotions (Powell and Butterfield, 1994). A perceived glass ceiling may thus create a confirmatory behavioral pattern that perpetuates gender inequality at the highest levels of power and decision-making.

Thus far, most research on the perceived glass ceiling was conducted in male-dominated organizations (e.g., finance, business management, law firms, science, and technology) and with a sole focus on women (e.g., Cech and Blair-Loy, 2010; Downes et al., 2014; Cohen et al., 2020; Babic and Hansez, 2021; but see Foley et al., 2002 for exception). These studies typically identify cultural (e.g., masculine work climate) and structural (e.g., family unfriendly policies) factors in male-dominated organizations as important antecedents of women’s glass ceiling perceptions. The current study adds to this knowledge base in several ways. First, we focus on work contexts where—on average—gender parity is achieved (i.e., in LSB fields), test whether women are perceptive of gender inequality in upward mobility in these fields, and whether sharp contrasts in gender representation at the top versus at lower ranks may, paradoxically, be even more pronounced in feminized LSB fields, relative to male-dominated NTE fields. Second, different from most other studies, we directly compare women academics to their male peers, to show how perceptions about the gender hierarchy in academia may differ depending on one’s gender identity, and to show how seeing a “thick” glass ceiling may have more detrimental consequences for women’s perceived career prospects toward leadership compared to men’s. Finally, in academia, there is a strong belief that career promotion and success hinges on meritocratic principles (i.e., individual ability; Cech and Blair-Loy, 2010), rather than contextual factors. As such, women in academia are often held individually accountable for their lower career success (e.g., women choose to “opt out” of ambitious careers themselves; Belkin, 2003). Our last goal is to refute this “choice rhetoric” (Vinkenburg et al., 2015), by testing the alternative hypothesis that women’s lower perceived career prospects toward leadership could also be explained by lower levels of career commitment among women compared to men.

### Theorizing and Hypotheses Formation

The glass ceiling can be defined as a structural, discriminatory barrier that women (but not men) face when advancing to the highest ranks in an organizational hierarchy. Compared to other forms of gender discrimination, the glass ceiling is a particular form of inequality following a specific set of criteria (see also Babic and Hansez, 2021). First, the glass ceiling refers specifically to *discrimination against women for leadership* positions and therefore exists beyond potential other gender differences in for example the level of education, tenure, experience, or skill (Cotter et al., 2001; Kulik and Rae, 2019). Second, the glass ceiling also refers to an *accelerating inequality*, meaning that the gender gap in men’s overrepresentation relative to women increases when moving further up to the higher echelons of management in an organization (Cotter et al., 2001). We see this in Dutch academia too such that relative to career progression at lower ranks (e.g., from PhD to assistant professor) women face the largest barriers when progressing from associate to full professor (i.e., the highest level; Figure 1; LNVH, 2018). Third, scholars agree that a glass ceiling is *difficult to establish objectively* or in absolute terms because the barriers that individual women face when trying to reach the highest levels of leadership are often intangible and difficult to attribute to gender discriminatory processes (Elliott and Smith, 2004; Babic and Hansez, 2021).

A large body of research has identified key antecedents of the glass ceiling in organizations for example, inadequate mentoring and network opportunities (Elacqua et al., 2009), a lack of transparency and fairness in performance criteria and promotion procedures (Lyness and Heilman, 2006) and differential treatment of women compared to men by upper management (Blau and Kahn, 2007; Kiaye and Singh, 2013). Moreover, academic cultures with a highly masculine vision of what successful leadership means (e.g., authoritarian, competitive, assertive, and individualistic; Van Vianen and Fischer, 2002; Babic and Hansez, 2021; Van Veelen and Derks, 2021) and with an ideal worker norm that is presumed incompatible with women’s work–life balance and care responsibilities (Morgenroth et al., 2021) contributes to women’s career stagnation and exit from academia. At the heart of these antecedents are biased-centered theories that argue that a glass ceiling exists because of (often unconscious) gender bias against promoting women for leadership positions.

Following social role theory, people hold gendered expectations about the roles men and women should fulfill in society (Eagly, 1987). Men are expected to be agentic “breadwinners” (e.g., assertive, ambitious, and competitive) and women are expected to be communal “homemakers” (e.g., modest, nurturing, and cooperative). We tend to associate leadership roles with the agentic characteristics we attribute more to men (Eagly and Karau, 2002; i.e., think manager and think male), while the communal characteristics we attribute more to women are seen as better suited for domestic roles (e.g., caregiver), and less fitting to ambitious leadership roles (Heilman and Parks-Stamm, 2007; Koenig et al., 2011). In the context of academia too, academics hold a highly agentic notion of the successful academic. Another project based on the same dataset (*N* ≈ 4,000 academics in the Netherlands) showed that irrespective of field or rank (i.e., assistant/associate/full professor), academics perceived the occupational stereotype of the successful academic as highly agentic (e.g., competitive, self-focused, and performance-oriented) while communal traits (e.g., collaborative, devoted teacher, and team player) were considered less important for academic success (Van Veelen and Derks, 2021). These findings point to the incompatibility between the agentic qualities deemed important for academic leadership on the one hand, and women’s gender identity being stereotyped as communal on the other. The incompatibly between gender and work roles likely contributes to women’s lower promotion probabilities in Dutch academia (van den Brink et al., 2010; Groeneveld et al., 2012) and suggests that academics are likely to see a glass ceiling, in the sense that in general, they see a contrast in women’s lower representation at the leadership level relative to the ranks below.

A key question is whether academics are still perceptive of the pervasive barriers that women face in advancing to leadership, when—on average—women have become well-represented in an academic field. One could argue that since women have become well-represented in LSB over the past decades, people have started to believe that gender bias in leadership is now becoming a thing of the past. In line with this idea, Kanter’s (1977) theory on tokenism would posit that as women become better represented in an organization, gender differences become less pronounced, gender stereotypes become less salient, and thus women’s promotion probabilities may increase. Recent empirical work also shows that in work contexts where the proportion of women is higher, women report to feel less stigmatized or discriminated against on the basis of their gender (Alt et al., 2019; Van Veelen et al., 2019). This could suggest that in the LSB fields, where women are well-represented overall, people might be less perceptive of the gendered status inequality that still exists in women’s representation in the academic hierarchy. By contrast, in the NTE fields, where gender ratios are highly skewed in favor of men, people may expect women to face more difficulty overall, and thus also in their upward career mobility. Indeed, arguments for a “thicker” perceived glass ceiling in male-dominated academic fields have been put forward in past research (Sanders et al., 2009; Groeneveld et al., 2012). However, their empirical evidence did not corroborate this idea, showing no effects of gender ratio in the field on female professors’ reported ease with which they obtained leadership themselves (Sanders et al., 2009), nor evidence for gender differences in female and male academics’ promotion probabilities depending on gender ratio of the field (Groeneveld et al., 2012).

In fact, we would argue that mere “strength in numbers” is likely not enough for gender bias in leadership to disappear in LSB fields—to the contrary. Empirical evidence on implicit associations shows that in sciences where the proportion of women has increased, the unconscious gender–science stereotype that favors men over women as being more fitting to a scientific career still prevails (Smyth and Nosek, 2015). Furthermore, in biological (i.e., life) sciences, where women are now a majority at undergraduate level, social network analysis provides evidence for gender bias in male undergraduates’ peer evaluations, with lower competence ratings attributed to female (versus male) students (Grunspan et al., 2016). In veterinary medicine, where women are now well-represented, experimental field research shows compelling evidence for gender bias, such that evaluators rated a male employee as more competent and more deserving of an—on average—8% higher salary than a female employee (Begeny et al., 2020). In psychological sciences too, while women are attracted in record numbers, gender gaps in pay, promotion, funding allocation, and eminence prevail (see Gruber et al., 2021 for an overview). In fact, with respect to NTE fields there is even evidence to suggest a *hiring advantage for women* over men (Ceci and Williams, 2015). Specifically, this experimental research showed a 2:1 preference for female compared to (equally qualified) male candidates for an assistant professor position. Furthermore, field research on the gender pay gap in Dutch academia suggests a higher gender pay gap in those fields where women are relatively well-represented (that is, lowest pay gaps were found in natural sciences and technology; De Goede et al., 2016). The latter findings are further substantiated by sociological research on labor market segregation in Europe showing that women are more likely to enter a leadership position in male-dominated compared to female-dominated occupations (Dämmrich and Blossfeld, 2017; Malin and Wise, 2018). Based on this evidence, we expect that despite being well-represented on average, academics will perceive the glass ceiling to be “thicker” in LSB compared to NTE sciences.

Furthermore, building from a social identity framework (Tajfel and Turner, 1979) we argue that female academics will be more perceptive of the thick glass ceiling in LSB sciences compared to their male peers. Social identities are those aspects of the self-concept that we derive from the groups we belong to, and that provide us with a sense of meaning and self-esteem (Abrams and Hogg, 1990; Brewer, 1991). Social identities acquire significance *via* the comparison of the ingroup with relevant outgroups (Turner et al., 1987), *via* internalization of ingroup norms and stereotypes (self-stereotyping; Spears et al., 1997; Van Veelen et al., 2016) and when in contexts where an ingroup’s status position is relatively low (Ely, 1995; Spears et al., 1997; Cadinu et al., 2013). Social identities drive our cognitions, emotions, and behaviors to the extent that these factors are salient in a given context (Hogg and Turner, 1987; Onorato and Turner, 2004). Women’s gender identity is generally one of the most chronically salient social identities in many contexts (Deaux et al., 1987). Specific to the academic context, the masculine culture (e.g., Bleijenbergh et al., 2013), the agentic stereotype of success (Van Veelen and Derks, 2021), and—particularly in LSB fields—the skewed representation of gender groups across academic ranks (Ceci et al., 2014; Figure 1), all make women’s gender identity highly salient and emphasize their low status position in academia. As such, for female (more than for male) academics their gender identity likely serves as a lens through which the social hierarchy in academia is perceived and understood (see Kteily and Richeson, 2016; Xiao et al., 2016 for a more in-depth discussion on how social identity shapes perception). Therefore, we expect female academics to be more perceptive of a glass ceiling such that, particularly in LSB sciences female academics are likely to see a sharper contrast in women being well-represented at the lower ranks yet underrepresented at the top, relative to their male peers.

Different from previous research, rather than operationalizing the perceived glass ceiling by directly asking people’s subjective opinions about whether they believe that in their organization women are disadvantaged in promotion for leadership relative to men (e.g., Foley et al., 2002; Elacqua et al., 2009; Downes et al., 2014; Cohen et al., 2020; Babic and Hansez, 2021), we introduce a novel, more indirect operationalization, namely, a perceived Glass Ceiling Index (GCI). We asked two separate questions: First academics were asked to think about the people in their direct working environment, and to estimate the ratio of women to men among their direct colleagues. Subsequently, academics were asked to estimate the gender ratio for at the full professor level in their department. We subtracted the perceived gender ratio at the colleague level from the perceived gender ratio at the top level (i.e., full professor level). This creates a GCI index where a score of 0 indicates similar gender representation at both levels, and a score GCI > 0 indicates a perceived glass ceiling (i.e., the proportion of women is lower in academic leadership relative to ranks below). The GCI is more indirect than other self-report measures in the sense that we did not directly ask participants to report on the difference in female representation at the leadership level versus at levels below themselves, but distilled this measure more indirectly. This indirect measure captures the perceived glass ceiling as a cognitive perceptual process rather than tapping into people’s, motivated belief systems.

There are several advantages to this GPI index relative to self-report glass ceiling scales used in prior research. First, items in perceived glass ceiling scales often conceptually conflate the extent to which people *perceive* a glass ceiling to exist, with their *beliefs* as to why it exists (Foley et al., 2002; Elacqua et al., 2009; Cohen et al., 2020; Babic and Hansez, 2021). Items in glass ceiling scales are for example “Do you believe that the glass ceiling exists in your company?”; “In my company, with equal experience and expertise, men have access to higher positions in the hierarchy than women”; “I believe our company is serious about eliminating barriers that prevent women from reaching their potential” (reverse-scored). This is problematic with regards to common method bias in cross-sectional research, because glass ceiling perceptions are often investigated in relation to self-reported differential treatment, gender discrimination and distributive justice measures (e.g., “promotion decisions [ ] in this organization are fair”; Foley et al., 2002; Babic and Hansez, 2021). The intercorrelations between these concepts are indeed very high in these studies (>0.80). Our GPI index focusses merely on the perceived size of the glass ceiling (i.e., how sharp is the contrast in gender representation at the top and below?), which ensures discriminant validity between glass ceiling perceptions and subsequent self-report measures about work and career-related variables. Second, since the GPI index does not directly refer to gender discriminatory practices, it circumvents socially desirable or biased answer tendencies, particularly observed among high-status groups (i.e., men), due to self-representational concerns or as a way of coping with negative topics, such as gender inequality by downplaying or denying its impact (Becker and Barreto, 2014; Scheepers and Ellemers, 2019). As such, the GCI index allows us to reliably interpret potential gender differences in glass ceiling perceptions as men and women observing a different *social reality* in the gender hierarchy in academia (rather than a different motivated response to interpret that reality). Taken together, we hypothesize that:

*H1:*Female academics in Life, Social and Behavioral fields (LSB) perceive a “thicker” glass ceiling toward leadership relative to their male peers and relative to academics in Natural Sciences, Technology and Economics (NTE).

#### Consequences of Seeing a Thick Glass Ceiling for Perceived Odds to Break Through It

While there are many studies on antecedents of the glass ceiling, relatively few investigate the relation between glass ceiling perceptions and how women and men perceive their own future career prospects (see Babic and Hansez, 2021). When female academics see a “thick” glass ceiling ahead of them, this likely goes hand in hand with lower estimated chances to break through the glass ceiling, and become a full professor themselves some day. Prior research on identity fit already shows that the more women in their early careers report lack of fit with a masculine occupational stereotype of success, the stronger their disengagement and turnover intentions from the field, for example in the royal navy (Peters et al., 2015) at the academy of royal surgeons (Peters et al., 2012); and among assistant professors in Dutch academia (Van Veelen and Derks, 2021). With respect to the perceived glass ceiling, what women in LSB fields see is that those who embody success are mostly male, while those who represent the rest of the field are mostly female. The observation that women are now becoming a numerical majority in LSB fields, yet men still predominantly hold positions of power and decision-making, sets a normative standard on who is to lead (men) and who is to follow (women; Braun et al., 2017). Particularly in academia’s *up-or-out* system where promotion practices are highly salient (Malos and Campion, 1995), this standard is likely discouraging for female academics’ perceived career prospects. Foley et al. (2002) showed in their study among ethnic minorities in law firms (also an up-or-out system) that self-reported glass ceiling perceptions were negatively related to perceived fairness of promotion decision outcomes in the firm. Building from this work we expect that for female academics in LSB fields, a higher glass ceiling index coincides with lower perceived career prospects to attain full professorship.

For male academics, glass ceiling perceptions are likely less impactful for their career prospects. Typically, high-status group members (i.e., men in academia) attribute less importance to their group identity and consider it less self-defining compared to low status group members (e.g., Spears et al., 1997; Cadinu et al., 2013). This would imply that in general, for male academics the gender ratio in their field, or variations therein across academic ranks, may have less implications for their own perceived career prospects. In addition, there is research to suggest that in feminized fields there is a male advantage in promotion for leadership, coined by the term the *glass escalator effect* (Williams, 1992). For example, men in female-dominated occupations (e.g., nursing) have been shown to report good relations with their—often male—supervisors (Williams, 1992; Allan, 1993), and to perceive their “male token status” as an advantage to hiring and promotion procedures (Evans, 1997; Kleinman, 2004; Torre, 2018). Men in feminized professions are also more often recruited for higher paying and higher status positions, even without actively searching for them (Kmec et al., 2010). Nevertheless, the empirical evidence for the glass escalator is not irrefutable, and contingent upon labor market changes (Price-Glynn and Rakovski, 2012; Williams, 2013). To this end, we arrive at the following hypotheses about gender differences in the consequences of seeing a thicker glass ceiling in LSB versus NTE fields:

*H2:*The thicker female academics perceive the glass ceiling to be in their field, the lower their estimated chances to become full professor themselves (while no effect or the reverse may be true for male academics).

Combining Hypothesis 1 and 2, we arrive at the following moderated mediation hypothesis, where we test the relationship between seeing a thicker glass ceiling in LSB compared to NTE sciences and women’s future career prospects in academia:

*H3:*Female (but not male) academics’ perception of a thicker glass ceiling in LSB compared to NTE fields explains their lower perceived chances to advance to academic leadership in LSB compared to NTE fields.

#### Alternative Explanations: Are Women in LSB Sciences Less Career-Committed?

The perceived glass ceiling effect offers a contextual explanation for women’s lower perceived promotion probabilities toward academic leadership, such that sharp perceived contrasts in women’s underrepresentation at the top versus overrepresentation at lower levels in LSB fields dampens women’s own leadership prospects at university. Oftentimes however, women are held individually accountable for their underrepresentation in leadership on the basis of their own (lack of) merit and career aptitude. Indeed, a common belief in academia (held by both men and women) is that academia is a meritocratic system and women just do not have the aptitude, motivation, or commitment required to attain full professorship as much as men do (Cech and Blair-Loy, 2010). Such rhetoric puts the onus on women for resolving gender gaps in pay and promotion in professional settings (Morgenroth and Heilman, 2017; Meeussen et al., 2021). We would contend that contextual barriers, rather than person-based career motivations, explain female academics’ lower perceived prospects to attain academic leadership (see also Cotter et al., 2001). Yet following research on career theory (Lent et al., 1999) it could well be true that aside from contextual conditions that support or hinder one’s career goals, personal factors such as a lack of career commitment account for women’s lower perceived odds to attain academic leadership relative to men’s. Career commitment refers to people’s “individual goal of advancing in their personal careers” (Ellemers et al., 1998, p. 718). Thus, apart from testing the effect of a perceived glass ceiling in LSB compared to NTE fields on male and female academics’ estimated odds to attain full professorship (Hypothesis 1–3), we also test the following alternative hypothesis:

*H_ALT:*Women’s individual levels of career commitment are lower than men’s, explaining their lower estimated chances to become full professor.

## Materials and MethodS

### Participants and Design

In the academic year 2017/18, 12.414 academic staff at assistant, associate, and full professor level from all 14 Universities in the Netherlands were invited to participate in an online survey called “Working in Academia.” A total of *N* = 4,295 academics completed the questionnaire (response rate of 35%). For the current investigation, the following inclusion criteria were applied: (1) participants who provided active informed consent or permission to use the data for scientific purposes (2) academics who self-identified as man or woman, (3) academics who were in the academic rank of assistant professor or associate professor (4) and academics who could be categorized in one of five classifications to indicate their scientific field as either highly math-intensive (NARCIS classification scheme,^2^ i.e., Natural Science and Technology^3^; Economics and Business) or non-math-intensive (i.e., Life Sciences, Social Sciences; Behavioral and Educational Sciences) following Ceci et al. (2014). After applying these criteria, *N* = 2,109 participants remained for further analyses (See Table 1 for Sample Characteristics).

**Table 1:** Sample Characteristics.

	Men	Women	Total
Age^a^ (chronological); *M (SD)*	45.37 (9.47)	41.92 (8.09)	43.87 (9.06)
Academic age^b^ (years since PhD); *M (SD)*	13.71 (8.31)	10.44 (6.65)	12.28 (7.79)
Rank ***N (%)***			
Assistant Prof.	739 (61.9%)	686 (74.9%)	1,425 (67.6%)
Associate Prof.	454 (38.1%)	230 (25.1%)	684 (32.4%)
Contract size^c^; *M (SD)*	38.34 (5.26)	37.06 (5.15)	37.70 (5.24)
Contract type^d^ ***N (%)***			
Permanent	917 (77.6%)	645 (71.5%)	1,562 (75.0%)
Fixed	265 (22.4%)	257 (28.5%)	522 (25.0%)
Academic discipline^e^ ***N (%)***			
Natural Sciences and Technology	501 (42.0%)	190 (20.7%)	691 (32.8%)
Economics	222 (18.6%)	101 (11.0%)	323 (15.3%)
Life Sciences	200 (16.8%)	158 (17.2%)	358 (17.0%)
Social Sciences	146 (12.2%)	197 (21.5%)	343 (16.3%)
Behavioral Sciences	124 (10.4%)	270 (29.5%)	394 (18.7%)

a *N* = 42 (2.0%) participants did not indicate their date of birth.

b *N* = 80 (3.8%) did not indicate their date of obtaining PhD.

c *N* = 132 (6.3%) did not indicate contract size.

d *N* = 25 (1.2%) did not indicate contract type.

e Note that the Dutch Medical University Institutes were not included in this investigation, because they have a different collective labor market agreement system compared to the Universities.

The sample consisted of *N* = 1,193 men (57%) and *N* = 916 women (43%). In terms of academic rank, *N* = 1,425 (68%) were assistant professor and *N* = 684 (32%) were associate professor. Among the women, 75% were assistant professor (relative to 62% of men) and 25% were associate professor (relative to 38% of men), signaling women’s underrepresentation at the higher rank. On average men in the sample were older (*M* = 45.37, *SD* = 9.47) than women (*M* = 41.92, *SD* = 8.09), *F*(1, 2065) = 79.14, *p* < 0.001, *η*^2^*_p_* = 0.036, also in terms of academic age (i.e., years since obtaining a PhD degree; *M_men_* = 13.71, *SD* = 8.31; *M_women_* = 10.44, *SD* = 6.65); *F*(1, 2027) = 91.87, *p* < 0.001, *η*^2^*_p_* = 0.043. Most academics (*N* = 1,562; 75%) held a permanent contract; women (29%) more often held a fixed-term contract than men (22%), *χ*^2^ (1) = 10.05, *p* = 0.002. The vast majority (*N* = 1,631; 83%) of academics worked fulltime (36 h a week or more); women more often held part-time contracts (*N* = 224, 27%) than men (*N* = 122, 11%), *χ*^2^ (1) = 84.98, *p* < 0.001. Finally, as stated before, academics from five academic disciplines were included in the sample^4^; the largest discipline represented in the sample was Natural Sciences and Technology (*N* = 691; 33%), followed by Behavioral and Educational Sciences (*N* = 394; 19%), Life Sciences (*N* = 358, 17%), Social Sciences (*N* = 343; 16%). The smallest discipline was Economics and Business (*N* = 323; 15%). Note that female and male academics were indeed not equally represented across disciplines (see Table 1).

The research had a cross-sectional design. In testing our hypotheses, our independent variables were gender (man/woman) and field [math-intensive (NTE) versus non-math-intensive (LSB)]. Since there are *a priori* differences in academics’ employment conditions across genders and fields (Tables 1, 2), in testing hypotheses on the perceived glass ceiling we included rank (assistant/associate professor), academic age (both linear and quadratic effects), contract type (permanent versus fixed-term) and contract size (hours per week) as covariates in the model. Our dependent variables where the perceived GCI, perceived chance to become a full professor and career commitment.

**Table 2:** Descriptive statistics model variables (*M, SD Pearson’s r*).

	*N*	*M*	*SD*	Pearson’s *r*												1	2	3	4	5	6	7	8	9	10	11	12
1. Academic age_linear_	2,109	12.28	7.65	1	0.961^***^	−0.049^*^	−0.466^***^	0.432^***^	−0.204^***^	−0.053^*^	0.082^***^	0.014	−0.068^***^	−0.309^***^	−0.041
2. Academic age_quadratic_	2,109	211.55	244.30		1	−0.049^*^	−0.370^***^	0.367^***^	−0.203^***^	−0.069^*^	0.084^***^	0.010	−0.073^***^	−0.338^***^	−0.045^*^
3. Contract size	2,109	37.80	5.08			1	0.023	0.071^**^	−0.117^***^	−0.113^***^	0.168^***^	0.034	−0.130^***^	0.149^***^	0.146^***^
4. Contract type^a^	2,109	0.26	0.44				1	−0.362^***^	0.067^**^	−0.075^***^	0.068^**^	0.069^**^	−0.012	−0.111^***^	−0.067^**^
5. Rank^b^	2,109	0.32	0.47					1	−0.137^***^	−0.083^***^	0.102^***^	0.033	−0.071^**^	0.207^***^	0.073^**^
6. Gender^c^	2,109	0.43	0.50						1	0.286^***^	−0.241^***^	0.032	0.260^***^	0.011	−0.019
7. Field^d^	2,109	0.52	0.50							1	−0.555^***^	−0.240^***^	0.337^***^	−0.109^***^	−0.065^**^
8. Gender Ratio_direct_	1926	3.28	0.73								1	0.391^***^	−0.642^***^	−0.108^***^	−0.095^***^
9. Gender Ratio_leadership_	1923	3.97	0.63									1	0.455^***^	0.070^**^	0.068^**^
10. GCI	1922	0.70	0.75										1	−0.047^*^	−0.035
11. %Chance Full Prof.	1916	41.53	31.66											1	0.352^***^
12. Career commitment	1923	3.57	0.90												1

Mean’s (*M*), Standard deviation’s (*SD*), and correlations (*Pearson’s r*). Covariates corrected for missing values

* *p* < 0.05;

** *p* < 0.01;

*** *p* < 0.001.

a Contract type: 0 = permanent; 1 = fixed-term.

b Rank level: 0 = assistant professor; 1 = associate professor.

c Gender: 0 = men; 1 = women.

d Field: 0 = Natural Sciences, Technology, and Economics (NTE); 1 = Life Sciences, Social Sciences and Behavioral Sciences (LSB).

### Procedure

The study was approved by the Ethics Committee of the Faculty of Social and Behavioral Sciences of the university (FETC17-010). Participants were approached *via* their university email address through the university’s internal HR communication system. The invitation was signed by either the rector or HR director of the university. The survey was available both in Dutch and in English and online for 2–3 weeks; after 1 week a reminder email was send out. Participants first provided informed consent, ensuring among others, anonymity, voluntary nature of participation, safety of data storage, the right to withdraw, and contact information, followed by questions about demographic and job characteristics. Then, questions about work circumstances (e.g., time for research, availability of resources) and professional self-perceptions and stereotypes were measured (Van Veelen and Derks, 2019, 2021), as part of the larger project. Subsequently, questions about career perceptions and future career opportunities in academia were answered as well as questions about the perceived gender ratio in the direct work environment and at the full professor level in the field. It took 15–20 min to complete the survey. Respondents were thanked for their participation but did not receive an actual reward.

### Measures

Below we report the measures in order of appearance in the survey. Note that our two questions regarding the gender ratio at different levels to calculate the GCI index measured completely at the end of the survey, after career commitment and estimated chances to become full professor. We did this to avoid priming effects that would make gender issues at work salient prior to measuring outcome variables. Herewith we further circumvent motivated response bias.

#### Math-Intensive vs. Non-intensive Field

Following Ceci et al. (2014), the five disciplines included in the current study are classified as either highly math-intensive (i.e., Natural Science and Technology, Economics; NTE) or non-math-intensive (i.e., Life Sciences, Social Sciences and Behavioral Sciences; LSB). We created a dichotomous variable to distinguish between highly math-intensive (NTE) and non-math-intensive fields (LSB).

#### Career Commitment in Academia

Two items measured career commitment, namely: “*I see my academic career as one of the most important things in my life*” and “*I consider it important to be successful in academia*” adapted from Ellemers et al. (1998). The inter-item correlation was high: *r* (1920) = 0.63, *p* < 0.001.^5^

#### Perceived Chances to Become a Full Professor

A one-item measure assessed perceived chances to become a full professor, namely: “*You indicated that you are currently an assistant [associate] professor. On a scale of 0–100%, how likely do you think it is that during your career you will become a full professor?*” Participants were asked to drag a slider to the percentage that would fit their answer best.

#### Perceived Glass Ceiling Index

We first asked academics to estimate the ratio of women relative to men in their direct work environment on a 5-point Likert scale (1 = only women, no men; 2 = mainly women, a few men; 3 = as many women as men; 4 = A few women, mainly men; 5 = no women, only men). Subsequently, we asked academics to make the same estimation on the same scale, this time about the gender ratio at the top level (i.e., full professor) in their department. We subtracted the perceived gender ratio in the direct work environment from the perceived gender ratio at the top level (i.e., full professor level). A GCI score of 0 indicates similar career advancement for men and women, a GCI > 0 indicates more difficulty for women to achieve the highest rank relative to men and a GCI < 0 indicates that it is easier for women to achieve the highest rank relative to men. The GCI could range from −4 to 4. For example, a score of 4 would indicate the thickest perceived glass ceiling possible, with a maximum contrast in the perceived proportion of women at the top rank (i.e., no women, only men; Likert score 5) relative to lower ranks (i.e., only women, no men, and Likert score 1).

### Analytical Strategy

Because it was possible for participants to skip questions they did not want to answer, we dealt with missing data. With respect to the covariates, we controlled for *a priori* gender differences in academic age, both the linear and the quadratic effect (i.e., years since receiving PhD; N_missing_ = 80; 3.8% of the data), for contract hours (N_missing_ = 132; 6.8% of the data), and for contract type (i.e., permanent versus fixed-term/other; N_missing_ = 25; 1.2%), academic rank (i.e., assistant or associate professor; no missing values). To avoid losing a substantial number of participants due to missing data on covariates in the statistical models, we imputed the mean of academic age and contract hours per week (0–40) for the missing cases and categorized missing cases for contract type in the category fixed-term/other. For the dependent variables, data loss due to attrition varied between 8.7 and 9.2%, and cases were deleted listwise, resulting in a sample size of *N* = 1908 to test the full hypothesized model.

Since we rely on cross-sectional self-report data in our design, we investigated the presence of common method variance by using Harman’s single factor test (Podsakoff et al., 2003). Here, all scale items [field, gender, gender ratio_directcolleagues_, gender ratio_leadership._ Perceived odds to become full professor (1 item), career commitment (2 items)] were entered in an unrotated exploratory factor analysis (PCA) with the number of factors constrained to one. Common method bias is assumed be to present when the single factor explains over 50% of variance. Yet our resulting factor merely explained 30% of variance in the items, ensuring that our concepts were independent, and ruling out potential problems with common method bias.

The statistical software program SPSS 27 was used to analyze the data. In a first step, we inspected descriptive statistics and correlations among model variables (Table 2). To test Hypothesis 1–3, a moderated mediation model (Model 58) was tested with PROCESS (Hayes, 2012). The macro uses ordinary least squares (OLS) analysis for calculating the mediation and moderated mediation effects, and bootstrapping for calculating the confidence intervals (CI). We used bias-corrected bootstrap CIs based on 5,000 bootstrap samples with a 95% level of confidence. When the confidence intervals do not include zero, the effect is interpreted as significant. The independent variable (X) was Field (NTE vs. LSB), the moderating variable (W) was Gender (men/women), the mediating variable (M) was Perceived GCI and the outcome variable (Y) was the Perceived Chance to become Full Professor. Academic Age (linear and quadratic), rank (assistant/associate professor), Contract Size (0–40 h a week), and Contract Type (permanent/fixed-term) were included as covariates.

Our sampling strategy was to obtain a sample size as large and representative for the population as possible. Because of this strategy, no *a priori* power analysis was conducted, but rather sensitivity analysis was conducted using G*Power software tool (Faul et al., 2007) to test the minimal effect size that would render statistical significance at conventional error probability levels (α = 0.05) to test our hypotheses (PROCESS moderated mediation Model 58; Hayes, 2012) given the sample size. In G*power (F-test family, regression analysis) we included 5 predictor variables (three main effects and two interaction terms: Field, Gender, GCI, Field x Gender, Gender x GCI) and 5 covariates (Academic Age (linear and quadratic), Rank, Contract Type, and Size), a minimal power requirement of 0.80, and a sample size of *N* = 1908, which demonstrated the ability to detect small effect sizes (*f*^2^ = 0.007) at 2.22 critical F-test ratios.

In additional analyses, we inspected whether an alternative mediation model, namely, that women in LSB sciences would be less career-committed and therefore estimate their chances to become a full professor to be lower, was a viable alternative model.

## Results

The current study investigated whether female (more than male) academics would perceive a thicker glass ceiling in non-math-intensive academic fields (LSB; where women are—on average—well-represented) compared to in math-intensive fields (NTE; where women are—on average—underrepresented; Hypothesis 1; moderation), whether for women (but not for men), perceiving a glass ceiling would lower their estimated odds to become full professor themselves (Hypothesis 2; moderation) and (combined), whether women’s (but not men’s) perception of a thicker glass ceiling in LSB compared to NTE fields would explain their lower perceived chances to advance to academic leadership (Hypothesis 3; moderated mediation). In addition, we tested the alternative hypothesis that rather than the perception of a glass ceiling (that is, a contextual explanation), women’s lower individual career commitment than men’s (person-based explanation), particularly in LSB sciences would mediate lower perceived chances to attain a leadership position.

### Descriptive Statistics

Descriptive statistics are displayed in Table 2. The perceived glass ceiling index was GCI_average_ = 0.70 (*SD* = 0.75). This shows that on average, the assistant and associate professors in this sample perceived a glass ceiling, such that women face more difficulty to progress to full professorship compared to men. Moreover, assistant professors perceived a thicker glass ceiling (GCI_assistant_ = 0.74; *SD* = 0.78) compared to associate professors (GCI_associate_ = 0.62; *SD* = 0.71), *t* (1293.94) = 3.23, *p* = 0.001, *CI_95%_* = 0.045; 0.186 (corrected for equal variances not assumed). Correlational data showed that the more precarious academics’ position was [that is, the more junior, *r* (1922) = −0.07, *p* = 0.003]; the lower in rank *r* (1922) = −0.07, *p* = 0.002; and the smaller the contract size, [*r* (1922) = −0.130, *p* < 0.001] the higher their perceived GCI. Resonating with Hypothesis 1, academics perceived the glass ceiling to be thicker in LSB (*M* = 0.94; *SD* = 0.77) compared to NTE disciplines (*M* = 0.43; *SD* = 0.65), *t* (1920) = −11.81, *p* < 0.001, *CI_95%_* = −0.576; −0.448. Moreover, women perceived a thicker glass ceiling (*M* = 0.92; *SD* = 0.77) than men (*M* = 0.53; *SD* = 0.70), *t* (1920) = −11.81, *p* < 0.001, *CI_95%_* = −0.463; −0.332.

Zooming in on the two levels of the gender ratio included in the GCI score (Table 2), academics saw more variation in the gender ratio in their direct work environment across field and gender compared to at the top (also evident from the correlational data). In NTE fields, both men (*M* = 3.70; *SE* = 0.02) and women (*M* = 3.69; *SE* = 0.04) reported to see overrepresentation of men in their direct work environment, *F*(1, 1922) = 0.051, *p* = 0.821, *η*^2^*_p_* < 0.001, in LSB fields men reported to see gender parity in their direct work environment (*M* = 3.02; *SE* = 0.03) and women in LSB fields reported to see a slight overrepresentation of women (*M* = 2.79; *SE* = 0.03), *F*(1, 1922) = 36.26 *p* < 0.001, *η*^2^*_p_* = 0.019. With regards to the gender ratio at the top, all academics scored around a 4 on the 5-point scale (i.e., perceiving mainly men at the full professor level). Women observed a slightly sharper overrepresentation of male full professors (*M* = 4.06; *SE* = 0.02) compared to men (*M* = 3.92; *SE* = 0.02; *F*(1, 1919) = 22.46, *p* < 0.001, *η*^2^*_p_* = 0.012), and the overrepresentation of men was perceived as more skewed in the NTE (*M* = 4.16; *SE* = 0.02) compared to the LSB (*M* = 3.82; *SE* = 0.02; *F*(1, 1919) = 135.80, *p* < 0.001, *η*^2^*_p_* = 0.066). The differences in the perceived gender ratio at the top were (significant, but) small and all boil down to the same conclusion; at the full professor level academics see a majority of men.

On average, academics in the sample estimated their chances to become full professor to be lower than chance, that is 41%. Moreover, assistant professors perceived lower chances (*M* = 37.07; *SD* = 29.29) compared to associate professors (*M* = 51.11; *SD* = 34.35), *t* (1034.41) = −8.72, *p* < 0.001, *CI_95%_* = −17.197; −10.877 (corrected for equal variances not assumed). Correlational data showed that the more precarious academics’ position was (that is the lower in rank *r* (1922) = 0.21, *p* < 0.001; having a fixed-term instead of permanent contract, *r* (1922) = 0.11, *p* < 0.001; and the smaller the contract size, *r* (1922) = 0.15, *p* < 0.001, the lower their perceived chances to become full professor were. There was no statistical evidence for gender differences in perceived chances to become full professor 1 day (*M_women_* = 41.92, *SD_women_* = 30.54; *M_men_* = 41.23; *SD_men_* = 32.51; *t* (1845.87) = −0.48, *p* = 0.632, *CI_95%_* = −3.532; 2.1442 (corrected for equal variances not assumed). In LSB fields, perceived chances to become a full professor were lower (*M* = 38.23, *SD* = 30.76) compared to NTE fields (*M* = 45.16, *SD* = 32.25), *t* (1876.47) = 4.81, *p* < 0.001, *CI_95%_* = 4.105; 9.765 (corrected for equal variances not assumed).

### Hypotheses Testing

**Figure 2:**
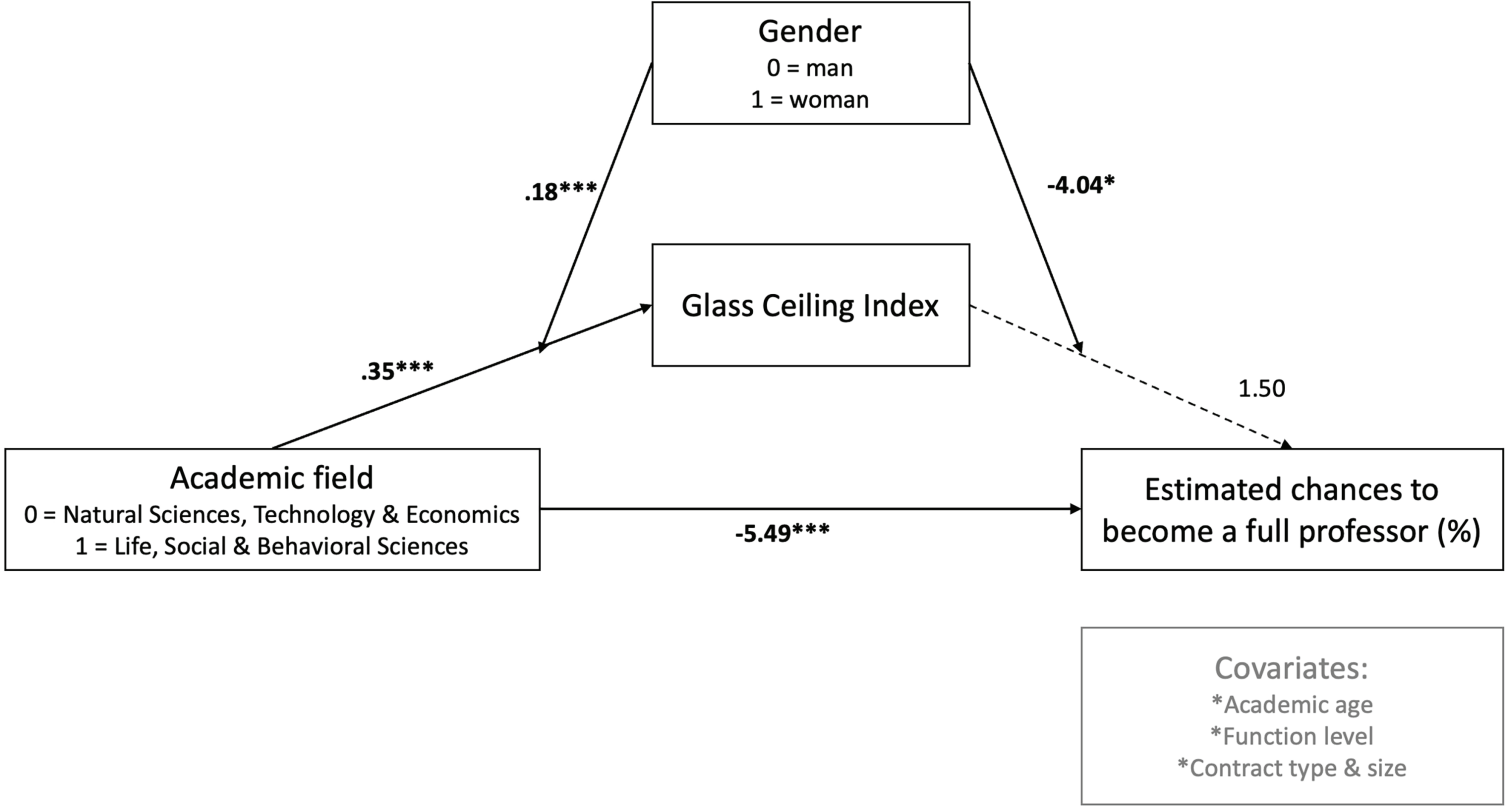
Moderated Mediation model (Model 58 Process) with Academic Field as predictor (X), the Glass Ceiling Index as mediator (M), Estimated Change to become full professor as dependent variable (Y) and Gender as Moderator (Z). Covariates are regressed on both M and Y.

**Figure 3:**
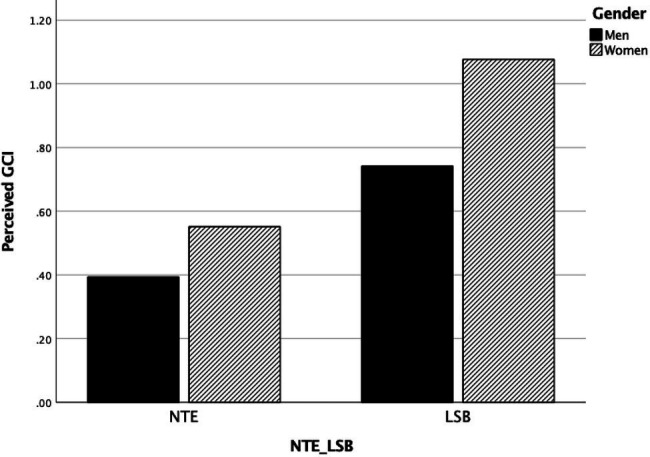
Two-way interaction effect Field (NTE vs. LSB) × Gender (Men vs. Women) on Perceived GCI.

Results of the moderated mediation model (Model 58, Hayes, 2012; Figure 2; Table 3) showed that with perceived GCI as an outcome variable, a main effect of Field was found, such that academics in LSB fields perceived a thicker glass ceiling compared to academics in NTE fields (*b* = 0.35, *SE* = 0.02, *p* < 0.001, *CI_95%_* = 0.262; 0.434). Moreover, the main effect of Gender revealed that female academics perceived a thicker glass ceiling compared to male academics (*b* = 0.16, *SE* = 0.05, *p* = 0.002, *CI_95%_* = 0.050; 0.259). These main effects were further qualified by a significant Field x Gender interaction (*b* = 0.18, *SE* = 0.07, *p* < 0.001, *CI_95%_* = 0.044; 0.311). Specifically, confirming Hypothesis 1 (see Figure 3) while both female (*b* = 0.53, *SE* = 0.05, *p* < 0.001, *CI_95%_* = 0.422; 0.628) and male (*b* = 0.35, *SE* = 0.05, *p* < 0.001, *CI_95%_* = 0.262; 0.434) academics perceived a thicker glass ceiling in LSB compared to NTE fields, the gender effect was more than two times larger in LSB (*b* = 0.34, *SE* = 0.05, *p* < 0.001, *CI_95%_* = 0.246; 0.424) compared to NTE fields (*b* = 0.16, *SE* = 0.05, *p* = 0.002, *CI_95%_* = 0.057; 0.259), with female academics in the LSB fields reporting the thickest glass ceiling: GCI_LSBFEMALE_ = 1.08.

**Table 3:** Moderated mediation results link between field, gender, perceived GCI and perceived odds to become full professor (*N* = 1908).

Moderated mediation results	Coefficient	SE	*95%* CI		Lower limit	Upper limit
*Outcome: Perceived GCI:* *R = 0.392, R^2^ = 0.154, F (8,1899) = 43.12, p < 0.001*		
Field	**0.348**	**0.044**	**0.262**	**0.434**
Gender	**0.158**	**0.052**	**0.057**	**0.259**
Field x Gender	**0.177**	**0.068**	**0.044**	**0.311**
Academic age (linear)	−0.001	0.009	−0.018	0.016
Academic age (quadratic)	<0.001	<0.001	−0.001	0.001
Contract size	−0.011	0.003	−0.018	−0.005
Contract type	−0.021	0.048	−0.109	0.067
Function level	−0.028	0.040	−0.106	0.050
*Outcome: Perceived odds (%) to full professor* *R = 0.521, R^2^ = 0.271, F (9,1898) = 78.446, p < 0.001*
Field	**−5.488**	**1.367**	**−8.170**	**−2.807**
GCI	1.495	1.203	−0.864	3.855
Gender	2.782	1.838	−0.823	6.387
GCI × Gender	**−4.025**	**1.698**	**−7.356**	**−0.694**
Academic age (linear)	−0.434	0.331	−1.073	0.226
Academic age (quadratic)	−0.048	0.010	−0.067	−0.029
Contract size	0.576	0.126	0.329	0.824
Contract type	4.004	1.736	0.599	7.409
Function level	26.999	1.537	23.984	30.013
*Conditional indirect effect at:*				
Men	0.521	0.431	−0.327	1.380
Women	**−1.329**	**0.674**	**−2.698**	**−0.066**
*Index of moderated mediation^a^*				
Gender	**−1.849**	**0.788**	**−3.439**	**−0.339**

Statistically significant effects (*p* < 0.05) for predictor variables are marked in bold.

a Difference between conditional indirect effects.

**Figure 4:**
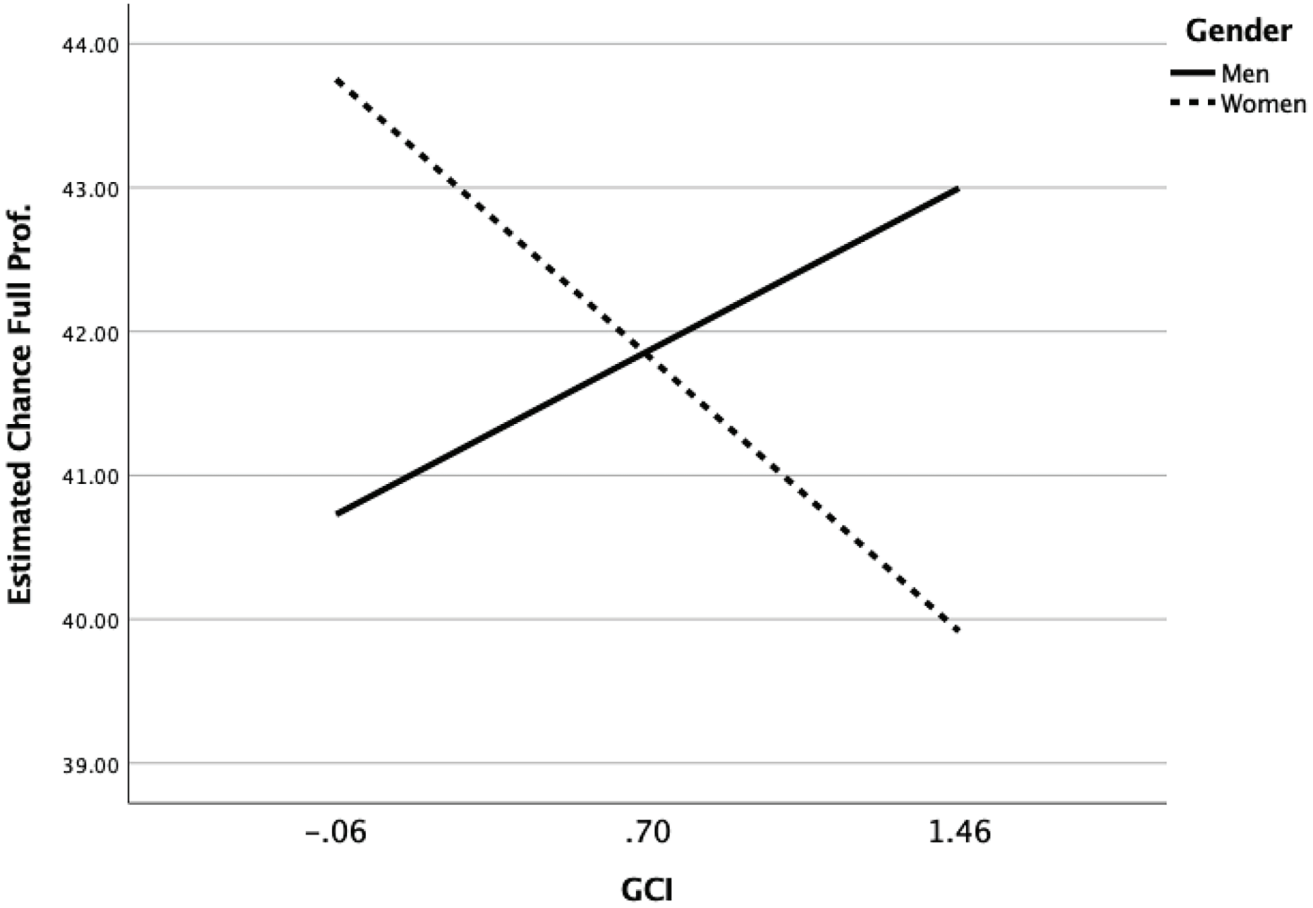
Interaction effect Perceived GCI × Gender on Perceived odds (%) to become full professor (interaction points plotted at −1 SD and +1 SD values from the mean GCI index).

Secondly, with regards to the perceived odds for academics to reach full professorship themselves, there was a main effect of Field such that the perceived odds to attain a full professorship position were lower in LSB sciences compared to NTE sciences (*b* = −5.49, *SE* = 1.37, *p* < 0.001, *CI_95%_* = −8.170; −2.806). While there were no significant main effects of Gender and perceived GCI on the odds to become full professor, there was a significant interaction effect of GCI × Gender (*b* = −4.04, *SE* = 1.69, *p* = 0.018, *CI_95%_* = −7.356; −0.694). Specifically, as depicted in Figure 4, for women, the estimated odds to become full professor dropped significantly as the perceived glass ceiling increased (*b* = 2.53, *SE* = 1.25, *p* = 0.044, *CI_95%_* = −4.991; −0.069). For men glass ceiling perceptions were not significantly related to estimated odds to become full professor (*b* = 1.495, *SE* = 1.20, *p* = 0.214, *CI_95%_* = −0.864; 3.855)—if anything, the data pattern was reversed for men. Confirming Hypothesis 2, this interaction pattern suggests that a thicker perceived glass ceiling in LSB compared to NTE fields will work to disadvantage women’s perceived chances of attaining academic leadership positions, but not men’s perceived chances.

Third, bootstrap results showed a conditional indirect effect of Field (i.e., NTE vs. LSB fields) on perceived odds to become a full professor through perceived GCI. Specifically, confirming Hypothesis 3, only for female academics, perceiving a thicker glass ceiling in LSB compared to NTE disciplines led to lower perceived odds to attain a full professorship position *via* a thicker perceived GCI (Indirect_women_: *b* = −1.33, *SE* = 0.67, *CI_95%_* = −2.698; −0.0657), while such indirect effect was not significant for men (Indirect_men_: *b* = 0.52, *SE* = 0.43, *CI_95%_* = −0.327; 1.380). Note that the difference between these conditional indirect effects was significant (Index = −1.85, SE = 0.79, *CI_95%_* = −3.490; −0.339).

Finally, to test the alternative hypothesis that career commitment would serve as a mediating variable to explain women’s lower perceived odds to attain academic leadership than men’s, we inserted career commitment into in our moderated mediation model 58. Results showed no main effect of Gender (*b* = −0.03, *SE* = 0.07, *p* = 0.665, *CI_95%_* = −0.156; 0.100) nor a significant interaction effect of Field x Gender (*b* = 0.07, *SE* = 0.09, *p* = 0.420, *CI_95%_* = −0.099; 0.238) on perceived career commitment. Thus, based on the current data, we reject the alternative hypothesis that perhaps women are less career-committed compared to men in LSB fields and compared to in NTE fields. And while higher career commitment did contribute to higher perceived odds to attain full professorship (*b* = 9.60, *SE* = 0.87, *p* < 0.001,*CI_95%_* = 7.891, 11.306), this was not contingent upon Gender (*b* = 1.45, *SE* = 1.32, *p* = 0.273,*CI_95%_* = −1.142, 4.044) nor did bootstrap tests show there was a conditional indirect effect of Field (NTE vs. LSB) on perceived odds to reach full professorship *via* career commitment, neither for women (Indirect_women_: *b* = −0.348, *SE* = 0.76, *CI_95%_* = −1.834, 1.137) nor men (Indirect_men_: *b* = −0.967, *SE* = 0.55, *CI_95%_* = −2.081, 0.097).

## Discussion

Most research on the careers of women in academia focus on the math-intensive fields natural science, technology, and economics (NTE), where women are vastly underrepresented. In this research, we shift focus on women’s academic careers in those fields where they have become well-represented: the life, social and behavioral sciences (LSB). Integrating theory on the glass ceiling (e.g., Cotter et al., 2001; Kulik and Rae, 2019; Cohen et al., 2020) with theory on social roles (Eagly, 1987) and social identities (Tajfel and Turner, 1979), we show that a mere strength in numbers does not shield women in LSB from perceiving gender inequality in women’s representation in leadership positions—to the contrary. Specifically, our data show that even though, on average, gender parity is achieved in LSB fields, female (more than male) academics perceive a thicker glass ceiling in LSB than in NTE fields. The sharper the perceived contrast in women being well-represented at lower levels, but less so at the top of academia, the lower female academics’ estimated chances to become full professor in LSB fields—a data pattern we do not see in male-dominated NTE fields, nor among male academics. Below we discuss implications and possible explanations for our findings.

### Theoretical Implications

By studying perceived glass ceiling effects among both male and female academics at mid-level careers sampled from the entire Dutch population of academics in LSB and NTE science fields, this research operates at a unique interface between social psychology, organizational science, and sociology. While inherently socio-psychological, in our literature review we relied on theory from all three disciplines to argue how gender roles and identities serve as a lens through which the social hierarchy in Dutch academia is observed, and how this shapes gender inequality in perceived career opportunities where we least expect it—in feminizing LSB fields. Such theoretical and empirical integration of theory to understand glass ceiling effects in academia is new and complements prior research that was unable to pinpoint the ambiguous relationship between gender ratios and promotion probabilities of women in academia (Sanders et al., 2009; Groeneveld et al., 2012). Specifically, our results show that in LSB fields, the *contrasts* that women see in the representation of men and women across academic ranks (rather than the gender ratio in general) introduce gender inequality in perceived career opportunities toward leadership.

With the current data, we can draw conclusions about how women’s *perceptions* about future career success in academia are likely shaped by the current gender hierarchy they see in their field. We cannot draw conclusions about how glass ceiling perceptions relate to women’s *actual* career advancement and success in academic fields. Why is it nevertheless important to learn what women’s (and men’s) career perceptions are in these fields? First, because we know from empirical studies that people’s estimated odds to successfully attain a leadership position in organizations relate to their career decisions, for example in terms of the willingness to make sacrifices for their career (Meeussen et al., 2021) their career adaptability (to flexibly deal with change or setback) and their turnover intentions (Ng and Feldman, 2014; Guan et al., 2015). So, lower expectations of future career success may translate into relatively more women at mid-level career stages deciding to quit academia, especially in LSB fields. Second, literature on career theory provides a strong empirical basis that subjective career success (perceptions about career success) and objective career success (pay, promotion) are interrelated (e.g., Poole et al., 1993; Ng et al., 2005; Abele and Spurk, 2009; Ballout, 2009). This could imply that female academics’ lower perceived odds to attain full professorship (cf. subjective career success) compared to men’s in LSB fields, may relate to other gender inequalities priorly observed in academia regarding objective career success (e.g., salary, research time, and resources) as (De Goede et al., 2016; Van Veelen and Derks, 2019). We think it is important to reveal these hidden cost of perceived glass ceilings for the careers of women in academia.

Our research focused on academics’ perceptions of women’s representation, and not on their *belief systems* about gender inequality in leadership. One interesting line of further inquiry would be to examine how glass ceiling perceptions relate to women’s beliefs about whether the current gender hierarchy in LSB and NTE fields is illegitimate or not. Given that women in NTE see a comparable underrepresentation of women around them as they see in positions of academic leadership, they may see the hierarchy as relatively open (permeable), and legitimate. However, although women in LSB are less likely to see the social hierarchy as permeable (because they see relatively few women at the top), this does not automatically mean that they will attribute the underrepresentation of women in leadership to gender discrimination, see it as illegitimate and fight for equal opportunities. Firstly, the narrative surrounding the social hierarchy in academia is one that is based strongly on meritocracy and individual mobility. Previous research had found that disadvantaged groups members are less likely to perceive group discrimination and to protest when meritocratic beliefs are activated (McCoy and Major, 2007; Jost et al., 2012). Secondly, the social setting in which men and women work together in LSB fields, with plenty of collaborative intergroup contact between the genders, is likely to undermine the likelihood that women will compare the outcomes of women to men’s and notice that their gender group may not be receiving equal opportunities (Saguy et al., 2009; Saguy and Chernyak-Hai, 2012). When members of disadvantaged groups perceive the social hierarchy as impermeable yet legitimate, they are less likely to work for social change (Ellemers et al., 1993). Instead, they will either opt out or work toward individual mobility and start perceiving themselves as very different from other women (e.g., self-group distancing, Van Veelen et al., 2020), which will leave the social hierarchy unchanged. Raising awareness that despite being well-represented, illegitimacies in women’s leadership advancement in LSB sciences are still prevalent is thus important.

Our study results lend further empirical support for the growing body of literature in social psychology showing that person-centered explanations for women’s lower promotion probabilities to leadership should be largely refuted. Instead, contextual explanations (e.g., a glass ceiling) grounded in biased-centered theories (e.g., role incongruency, Eagly and Karau, 2002; social identity; Tajfel and Turner, 1979) form a more solid evidence base as to why women still face disproportionate barriers in attaining leadership relative to men (e.g., Ellemers, 2014; Van Laar et al., 2019; Meeussen et al., 2021; Morgenroth et al., 2021). Similar trends are observed in literature in organization science where career theorists’ have been critiqued on their overemphasis on individual agency as important parameters to predict subjective and objective career success, and neglecting the role of contextual issues (Evetts, 1992; Brown, 2002). Our study findings contribute to the growing consideration of the organizational, societal and political context in gendered career trajectories (Mayrhofer et al., 2007; Järlström et al., 2020). It is not only how women see themselves, in terms of their own career commitment, but also how they see the social hierarchy in academia and how their gender identity is reflected in that hierarchy, that accentuates their low status position relative to men’s. Gender differences in the perceptiveness to that invisible glass ceiling explain women’s lower estimated chances to reach full professorship relative to men in LSB fields, not gender differences in career commitment.

The glass ceiling metaphor suggests that this is a barrier that can be broken or shattered (Kulik and Rae, 2019). As evidenced from our research however, a glass ceiling is not broken when a small group of women achieves the highest levels of academic leadership. Intuitively, a “broken” glass ceiling would mean that once women are entering leadership positions, there are more opportunities for women who follow. This is not the case in LSB sciences. Specifically, as research on the queen bee phenomenon shows, women who have made it to top positions have had to make many sacrifices to attain that position, and have often socialized to “become one of the boys” themselves in order to fit to an agentic leadership prototype (Faniko et al., 2017, 2021). As such, women who paved the way toward academic leadership may not necessarily be advocates of social change. In future research, a further investigation of the role of gender identification in relation to glass ceiling perceptions in academia would be valuable. Experiences of gender discrimination in the work context are more strongly felt by women who strongly identify with their gender (Ellemers et al., 2002). In response to such discrimination, women who identify strongly with their gender are more likely to advocate for social change and to fight for equal rights, while less gender identified women are more likely to dissociate from, downplay or even deny issues with gender discrimination (Derks et al., 2016; Britton, 2017). Potentially, this latter individual mobility strategy has thus far been more fruitful for women to attain academic leadership. Further insight in social identity coping mechanisms in relation to views from below (e.g., assistant/associate professor) and above (e.g., full professors) the glass ceiling would deepen our understanding as to what motivates women (and men) to break glass ceilings and why women would opt for individual mobility to attain leadership, slipping through the cracks of the glass ceiling, rather than breaking it altogether.

### Strengths, Limitations, and Practical Implications

A strength of our research is our new Glass Ceiling Index (GCI). Different from prior self-report measures we did not directly ask participants to estimate or interpret the difference in women’s representation at the top relative to at lower ranks. Instead, we asked two questions about the gender ratio in the direct work environment and in leadership, and we did so at the very end of the survey. Therefore, even though academics in our sample were not actively made aware of gender discriminatory practices in leadership in their field, women’s lower perceived chances to become full professor in LSB fields were nevertheless significantly related to the indirect observation of a “thick” glass ceiling. It thus seems that contextual factors that subtly signal women’s unequal opportunity toward leadership thus inform women about their potential leadership success. Recent research shows how contextual cues that signal lower odds for women to attain leadership explain women’s lower willingness to make sacrifices for their careers relative to men’s (Meeussen et al., 2021). Thus, rather than a matter of individual choice, women’s lower perceived opportunities and subsequent choices about leadership advancement are more likely the result of an informed decision-making process. As evidenced in our study, for female academics in LSB fields, a contextual constraint informing their future career prospects is their less opportune position in the status hierarchy relative to men’s.

Our GCI index included two parameters, the perceived gender ratio in the direct environment and at the top, and from the *contrast* between the two we distilled the size of the perceived glass ceiling. In terms of interpretation of our findings, a thicker perceived glass ceiling in LSB (but not NTE) fields can be understood as women seeing a “lack of female leadership” as well as women seeing a “reservoir of women” stuck ad mid-level careers. With respect to the latter, one could speculate that apart from a “glass ceiling” other metaphors in the literature about “sticky floors” (Morgan, 2015) and “frozen middles” (McKinsey, 2012) may also apply to women in LSB fields. Glass ceilings, sticky floors or frozen middles can be regarded as similar since they all focus on barriers women face in upward mobility toward leadership (Shabsough et al., 2021). Yet the driving forces behind them may be different. A glass ceiling metaphor suggests that women are “pushed away” from leadership positions, while the sticky floor or the frozen middle suggests women being “pulled back” into low or middle management positions with lower pay and lower mobility for a longer period of time (Smith et al., 2012; Carli and Eagly, 2016). With regards to practical implications, in addition to a “think manager-think male” analogy to understand women’s lower perceived propensity to attain leadership in feminized, social science fields, it is important to also take into account a “think follower-think female” analogy (Braun et al., 2017). Indeed, female academics are more often than men considered the “communal colleagues” the “devoted teachers” and more often receive requests for “administrative/non-promotable tasks” (Vesterlund, 2015; Babcock et al., 2017). In designing policy interventions to break gendered barriers toward academic leadership in LSB fields, universities should thus not only focus on reducing existing stigma and bias surrounding women’s competence for leadership, but also focus on ensuring that women are not overburdened with non-promotable tasks that make it more difficult to self-promote, to stand out and to be noticed for leadership.

Increasingly, universities have diversity programs in place to facilitate (gender) diversity and inclusion of academic staff. Yet most diversity programs are only targeted at the influx of employees (Dobbin and Kalev, 2016; Vink et al., 2021, unpublished). For example, affirmative action programs or anti-bias trainings during recruitment and selection procedures explicitly aim to invite more women in positions at the point of entry in the academic pipeline (e.g., in a tenure track position and/or as assistant professor). Far fewer diversity measures follow-up on entry programs to ensure equitable promotion and retention of employees further up the career ladder (Bokern et al., 2021). While policies targeted at influx might (still) be fitting in male-dominated NTE fields, our study results inform us that particularly for women in LSB, a follow-up plan should be in place further up the academic pipeline to ensure that women see equal opportunities in their promotion for leadership relative to their male peers. On a symbolic level, one example of how to showcase more inclusive exemplars of (women in) academic leadership, is an initiative by a Dutch University who included 99 portraits of female professors on the walls of the Senate Chamber that originally contained 117 portraits of men and one woman (Athena’s Angels, 2016), Visibility of women in academic leadership may help early career female academics to envision themselves in a full professor position.

On an institutional level, our study results contribute to current debates about the implementation of the gender quota in (academic) leadership. Gender quota have been shown to increase female representation in the board rooms, yet there is little evidence for spill-over to other areas of leadership (Wang and Kelan, 2013; Wauters et al., 2014; Geys and Sørensen, 2019). While diversity quota may not have the anticipated immediate trickle-down effects many institutions hoped for, this research shows that on a psychological level gender quota are likely to serve an important function for early career female academics perceived future career prospects. Our study results suggest that doing nothing about the skewed gender representation across academic ranks in LSB fields does negate women’s perceived opportunities to career advancement in academia—something that may be avoided when gender quota are in place.

With a unique sample of around 2,000 academics at mid-level careers in the Netherlands the ecological validity of our field data is high. What’s more, the investigation of perceptions of a glass ceiling in academia likely forms a powerful parameter in the psychology of junior and mid-level female academics and how they see and act with regards to their future career at university. While both our sample and psychological approach are unique, there are several limitations to the data. First, as pointed out above, the current data showed a negative relationship between women’s GCI and their *perceived* odds of advancing to leadership, but we have no data on women’s *actual* leadership advancement in academic fields where a “thick” glass ceiling is observed. To further substantiate and validate the importance of these findings, studying women’s actual career behaviors in relation to their perceptions is pertinent. Such research could empirically corroborate whether seeing a glass ceiling ahead indeed act as a self-fulfilling prophecy with women (self-) selecting out of academia (Powell and Butterfield, 1994).

Second, due to the cross-sectional nature of the data, claims of causality should be made with caution. We could quite safely assume that relatively stable parameters (Field, Gender, Gender Ratio) are likely to correlate strongly with women’s perception of a glass ceiling, and as such *precede* women’s perceived odds to progress to full professorship. Also, in terms of third variable explanations, by inserting covariates (i.e., academic age, tenure, rank, and contract) we were at least partially able to rule out that gender differences found in perceived glass ceilings and career prospects are attributable to those aspects on which female and male academics at mid-level careers already differ. Nevertheless, also in relation to the previous point, only longitudinal data, following mid-level career academics as they transition to new career phases would allow for making actual claims about the effects of perceived gender differences in odds to advance to leadership, for example based on survival analyses techniques. Third, self-report data in the study may raise concerns about common method bias (Podsakoff et al., 2003), yet scale testing demonstrated that such was negligible. Moreover, a key element of our model was to test for moderation (e.g., Field x Gender), and moderation effects cannot be artifacts of common method bias (Siemsen et al., 2010). Finally, because this research was conducted in the Netherlands, a country that scores relatively low on female representation in academic leadership relative to other European countries (European Commission, 2019) we cannot generalize our findings to other countries. In future research, cross-cultural comparisons, for example connecting glass ceiling effects to endorsement of gender–science stereotypes across fields and nations will be valuable.

## Conclusion

In the life, social and behavioral fields, women’s representation has grown rapidly over the past decades such that, on average, gender parity is almost achieved. Therefore, gender issues are seemingly less at stake in these fields, compared to the male-dominated natural sciences, technology, and economics. The results from this research suggest that women’s higher numerical representation in LSB fields does not negate a masculine normative standard about academic leadership and success—to the contrary. Compared to in NTE fields, women at mid-level careers in LSB sciences reported to perceive a thicker glass ceiling, such that they saw a sharper contrast between women being well-represented at the lower, yet underrepresented at the top positions. This sharper contrast was negatively related to women’s, but not men’s, estimated odds to become a full professor some day; a gender inequality we did not observe in NTE fields. We conclude that women assistant and associate professors in LSB deal with gender discrimination toward full professorship, perhaps more so than women in NTE fields do. For this awareness should be raised and tailor-made policy interventions should be designed.

## Data Availability Statement

The datasets presented in this article are not readily available because the data is not publicly available due to privacy or ethical restrictions, e.g., containing information that could compromise the privacy of research participants. Therefore, only the Principal Investigators have access to the raw datafiles. The anonymized dataset to support the findings of this study are available upon reasonable request from the corresponding author. Requests to access the dataset should be directed to r.vanveelen@uu.nl.

## Ethics Statement

The studies involving human participants were reviewed and approved by Ethics Committee of the Faculty of Social and Behavioral Sciences Utrecht University (FETC17-010). The patients/participants provided their written informed consent to participate in this study.

## Author Contributions

RV contributed to conceptualization, formal analysis, investigation, methodology, project administration, visualization of data, writing—original draft, and writing—review and editing. BD contributed to conceptualization, funding acquisition, methodology, project administration, supervision, and writing—review and editing. All authors contributed to the article and approved the submitted version.

## Funding

This research was supported by funding from the Dutch Network of Women Professors (non-commercial) awarded to BD, and by an NWO VIDI grant (016.155.391) awarded to BD.

## Conflict of Interest

The authors declare that the research was conducted in the absence of any commercial or financial relationships that could be construed as a potential conflict of interest.

## Publisher’s Note

All claims expressed in this article are solely those of the authors and do not necessarily represent those of their affiliated organizations, or those of the publisher, the editors and the reviewers. Any product that may be evaluated in this article, or claim that may be made by its manufacturer, is not guaranteed or endorsed by the publisher.

## Footnotes

^1^Natural Sciences and Technology are also often abbreviated with the term STEM (Science, Technology, Engineering and Math; see also Ceci et al., 2014).

^2^ www.narcis.nl

^3^Note that in the NARCIS classification of academic disciplines in the Netherlands, Natural Sciences and Technology are grouped into one category.

^4^Participants from the disciplines Humanities and Law & Governance were not included in the analysis, as they do not fall within the scope of Ceci et al.’s (2014) classification of sciences as math-intensive (NTE) and non-intensive (LSB).

^5^We are aware that the dependent variables in our model comprised only one or two items and that, psychometrically, using multiple items with validated scales is preferable. Yet we had a unique opportunity to access the entire Dutch population of (tenured) academics in the Netherlands. Thus for practical reasons (i.e., to ensure a large participation rate and by reducing participant burden) we needed to keep the online questionnaire as short as possible. Hence, we were very limited in the number of items we were able to include per variable. Given these practical constraints, there is a good case to make for including single-item measures for psychological constructs (like with the perceived chance to become a full professor on a slider scale from 0 to 100%; see also Fisher et al., 2016; JOHP). Specifically with regards to career commitment, the original scale (Ellemers et al., 1998) consisted of 6 items. We selected the two items that, based on scale validity analyses, showed highest factor loadings (>0.80) across the studies that tested the psychometric quality of this scale. The two items thus form the core elements of the psychological construct career commitment.
